# Biofeedback for Motor and Cognitive Rehabilitation in Parkinson’s Disease: A Comprehensive Review of Non-Invasive Interventions

**DOI:** 10.3390/brainsci15070720

**Published:** 2025-07-04

**Authors:** Pierluigi Diotaiuti, Giulio Marotta, Salvatore Vitiello, Francesco Di Siena, Marco Palombo, Elisa Langiano, Maria Ferrara, Stefania Mancone

**Affiliations:** Department of Human Sciences, Society and Health, University of Cassino and Southern Lazio, 03043 Cassino, Italy; giulio.marotta@unicas.it (G.M.); salvatore.vitiello@unicas.it (S.V.); francesco.disiena@unicas.it (F.D.S.); marco.palombo@unicas.it (M.P.); elisa.langiano@unicas.it (E.L.); m.ferrara@unicas.it (M.F.); s.mancone@unicas.it (S.M.)

**Keywords:** biofeedback, Parkinson’s disease, motor rehabilitation, neurofeedback, electromyography, heart rate variability, cognitive Enhancement, non-pharmacological therapy, wearable neurotechnology, autonomic regulation

## Abstract

(1) Background: Biofeedback and neurofeedback are gaining attention as non-invasive rehabilitation strategies in Parkinson’s disease (PD) treatment, aiming to modulate motor and non-motor symptoms through the self-regulation of physiological signals. (2) Objective: This review explores the application of biofeedback techniques, electromyographic (EMG) biofeedback, heart rate variability (HRV) biofeedback, and electroencephalographic (EEG) neurofeedback in PD rehabilitation, analyzing their impacts on motor control, autonomic function, and cognitive performance. (3) Methods: This review critically examined 15 studies investigating the efficacy of electromyographic (EMG), heart rate variability (HRV), and electroencephalographic (EEG) feedback interventions in PD. Studies were selected through a systematic search of peer-reviewed literature and analyzed in terms of design, sample characteristics, feedback modality, outcomes, and clinical feasibility. (4) Results: EMG biofeedback demonstrated improvements in muscle activation, gait, postural stability, and dysphagia management. HRV biofeedback showed positive effects on autonomic regulation, emotional control, and cardiovascular stability. EEG neurofeedback targeted abnormal cortical oscillations, such as beta-band overactivity and reduced frontal theta, and was associated with improvements in motor initiation, executive functioning, and cognitive flexibility. However, the reviewed studies were heterogeneous in design and outcome measures, limiting generalizability. Subgroup trends suggested modality-specific benefits across motor, autonomic, and cognitive domains. (5) Conclusions: While EMG and HRV systems are more accessible for clinical or home-based use, EEG neurofeedback remains technically demanding. Standardization of protocols and further randomized controlled trials are needed. Future directions include AI-driven personalization, wearable technologies, and multimodal integration to enhance accessibility and long-term adherence. Biofeedback presents a promising adjunct to conventional PD therapies, supporting personalized, patient-centered rehabilitation models.

## 1. Introduction

Parkinson’s disease (PD) is a progressive neurodegenerative disorder primarily characterized by the loss of dopaminergic neurons in the substantia nigra pars compacta, leading to significant disruptions in the cortico–basal ganglia–thalamic circuitry. This neuronal degeneration results in a cascade of motor and non-motor impairments that progressively impact patients’ quality of life and functional independence. The hallmark motor symptoms of PD include bradykinesia, rigidity, resting tremor, and postural instability, which are the primary criteria for clinical diagnosis [1,2,3]. These motor deficits often lead to increased fall risk, impaired gait, and reduced mobility, significantly compromising daily activities and increasing the burden of care for both patients and caregivers [4,5,6,7]. However, beyond classical motor impairments, non-motor symptoms (NMSs) such as cognitive decline, emotional dysregulation, sleep disturbances, autonomic dysfunction, and fatigue are also prevalent in PD and, in some cases, may precede motor symptoms by years [4,8,9]. These NMSs are often underdiagnosed yet contribute substantially to disease burden and disability, affecting emotional well-being, cognitive flexibility, and social participation [10,11,12].

Despite the advancements in PD management, the available pharmacological treatments, such as levodopa, dopamine agonists, and monoamine oxidase-B (MAO-B) inhibitors, primarily provide symptomatic relief but do not halt the underlying neurodegenerative process [13,14]. Over time, the effectiveness of levodopa diminishes, leading to the emergence of motor fluctuations and dyskinesias, making long-term symptom control increasingly challenging [15,16]. Deep brain stimulation (DBS) has emerged as an effective neurosurgical intervention for patients with advanced PD, particularly for managing medication–refractory motor fluctuations and tremor [17,18,19]. While DBS has demonstrated substantial benefits in improving motor function and quality of life, it remains an invasive procedure with potential complications, and not all PD patients are eligible candidates due to their cognitive and psychiatric comorbidities [20,21]. These limitations underscore the urgent need for complementary, non-invasive therapeutic approaches that can be integrated into standard PD care to enhance motor function, cognitive resilience, and autonomic stability. In this context, biofeedback has emerged as a promising rehabilitation approach, leveraging real-time sensory feedback to enable patients to gain voluntary control over physiological processes [22,23,24].

Biofeedback is based on the principles of operant conditioning, where individuals receive continuous feedback on physiological signals (e.g., muscle activity, heart rate variability, or neural oscillations) and learn to modulate these signals through repeated practice [25,26]. By training patients to self-regulate their physiological responses, biofeedback interventions offer a non-invasive strategy to optimize motor performance, autonomic function, and emotional well-being. Over the past two decades, several biofeedback modalities have been explored in PD rehabilitation, with varying degrees of clinical success. Among the most studied biofeedback techniques in PD are:

- Electromyographic (EMG) biofeedback, which facilitates voluntary control over muscle activity by providing real-time visual or auditory feedback on electromyographic signals. This method has been investigated for its potential to reduce rigidity, improve postural stability, and enhance motor coordination in PD patients [27,28,29,30,31].
- Heart rate variability (HRV) biofeedback, which targets autonomic nervous system (ANS) dysregulation by training patients to modulate their breathing patterns to influence heart rate variability and vagal tone. This approach has shown promise in reducing PD-related anxiety, depression, and orthostatic hypotension [32,33,34].
- Electroencephalographic (EEG) neurofeedback, which focuses on training individuals to modulate dysfunctional brain oscillations associated with motor impairment, executive dysfunction, and cognitive decline. EEG–NF interventions have demonstrated potential for improving motor coordination, cognitive flexibility, and emotional self-regulation in PD [35,36,37].

Each of these biofeedback modalities targets distinct aspects of PD pathology, providing a multimodal and personalized approach to disease management. Given the heterogeneous nature of PD symptomatology, biofeedback-based rehabilitation has the potential to address both motor and non-motor domains, offering a holistic, patient-centered intervention. However, despite the growing body of evidence supporting biofeedback applications in PD, several challenges remain, including the lack of standardized protocols, variability in patient responsiveness, and the need for large-scale clinical validation [38,39].

The objective of this review is to provide a comprehensive and updated synthesis of biofeedback and neurofeedback interventions for Parkinson’s disease (PD), focusing on their clinical effects, feasibility, and future potential. Specifically, the review aims to (1) summarize and compare the therapeutic outcomes of EMG-, HRV-, and EEG-based biofeedback protocols for both motor and non-motor symptoms in PD; (2) evaluate the clinical applicability of these techniques in real-world settings; and (3) identify trends, limitations, and opportunities for future research and implementation.

Biofeedback techniques have shown general benefits in neurological rehabilitation by promoting self-regulation, enhancing neuroplasticity, and enabling non-invasive, patient-centered care. In the context of PD, biofeedback is particularly valuable for addressing complex motor dysfunctions (e.g., bradykinesia, rigidity, and gait abnormalities) and non-motor symptoms such as dysautonomia, anxiety, and cognitive decline.

Previous narrative reviews on this topic have either focused on a single modality or lacked systematic comparisons across feedback types. For example, some surveys have emphasized neurofeedback in cognitive training, while others have reviewed EMG biofeedback in physical therapy settings. This review builds upon those efforts by offering an integrated perspective that compares multiple modalities across clinical domains.

Methodologically, this review adopts a structured narrative approach, using predefined criteria for article selection, categorization of intervention types, and analysis of clinical outcomes by functional domain (motor, autonomic, and cognitive). In contrast to previous reviews, our synthesis emphasizes translational potential, feasibility for home use, and the integration of AI-driven or multimodal systems, thus offering both a broader scope and a more clinically oriented framework.

With continued advancements in biofeedback technology, wearable devices, and artificial intelligence-driven adaptive training, biofeedback holds the potential to become a cornerstone of non-pharmacological PD management, complementing existing therapies and improving functional independence, cognitive resilience, and overall quality of life for individuals living with PD.

## 2. Methods

A comprehensive literature search was performed across PubMed, Scopus, and Web of Science to identify relevant peer-reviewed studies published from January 2010 to April 2025. Search terms included combinations of: “Parkinson’s disease” OR “PD” AND “biofeedback” OR “neurofeedback” OR “EEG” OR “EMG” OR “HRV” OR “visual feedback” OR “auditory feedback” OR “vibrotactile feedback.” Additional sources were retrieved by screening reference lists of included studies.

Studies were considered eligible if they met the following criteria: if they were peer-reviewed publications in English that involved human participants diagnosed with PD, interventions based on biofeedback or neurofeedback, and quantitative outcomes reported in at least one clinical domain (motor, cognitive, and autonomic). Exclusion criteria included case reports, editorials, narrative reviews without original data, and studies not focused on PD or reporting measurable outcomes.

After duplicate removal, titles and abstracts were screened to assess relevance. Full texts of potentially eligible articles were retrieved and assessed based on inclusion and exclusion criteria. The selection process was performed independently by two reviewers, with discrepancies resolved by consensus. A total of 15 studies met all inclusion criteria and were included in the final synthesis.

Data from each study were extracted into a standardized template including authorship and year, sample size and characteristics, biofeedback modality used (EMG, HRV, EEG, and multimodal), intervention duration, study design, and reported outcomes across clinical domains. Studies were descriptively grouped by feedback type and analyzed based on motor, autonomic, and cognitive outcome patterns.

Quality appraisal was performed based on study design (e.g., randomized controlled trial, pilot study, or controlled trial), clarity in reporting methods and outcomes, sample size adequacy, and presence of control conditions. The methodological diversity of included studies was acknowledged, and no formal meta-analysis was conducted.

**Figure 1:**
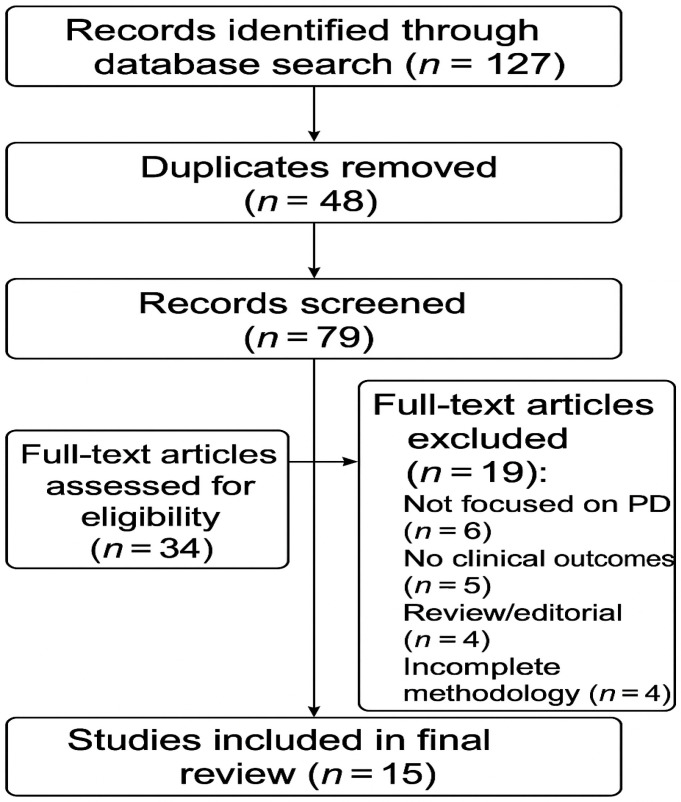
PRISMA flow diagram illustrating the study selection process for the review. The diagram shows the number of records identified through database searching that were screened for eligibility, excluded with reasons, and finally included in the qualitative synthesis (*n* = 15 studies).

To increase transparency, the study selection process is illustrated through a PRISMA-style flow diagram (Figure 1), outlining the number of records identified, screened, excluded, and included in the final review.

A summary of the key studies included in this review is provided in Table 1. The table outlines the authors, sample characteristics, type of biofeedback intervention, duration of the intervention, main outcomes, and study design for each study analyzed.

## 3. Results

### 3.1. Effects of EMG Biofeedback on Motor Outcomes in Parkinson’s Disease

Electromyographic (EMG) biofeedback has been shown to enhance motor function in individuals with Parkinson’s disease (PD) by enabling voluntary control over muscle activation through real-time feedback [53]. This modality addresses key motor impairments such as bradykinesia, rigidity, and postural instability [30,45] by retraining sensorimotor coordination and promoting more efficient muscle activity. EMG biofeedback helps reduce the co-contraction of antagonistic muscle groups and facilitates smoother, more coordinated movement. Studies demonstrate improvements in gait parameters, such as increased stride length, reduced gait variability, and enhanced walking speed [44]. EMG biofeedback has also been applied to dysphagia management, with evidence showing that real-time feedback during swallowing exercises improves long-term functional oral intake and reduces dysphagia severity [32]. Additional modalities such as vibrotactile [41,46], auditory [40,54], and visual biofeedback [42] have further demonstrated efficacy in improving gait, posture, and balance through real-time sensory cueing.

### 3.2. Effects of EMG Biofeedback on Non-Motor Outcomes

Although primarily designed for motor rehabilitation, EMG biofeedback has also been applied to non-motor symptoms, particularly voice disorders. For example, combining EMG biofeedback with virtual reality and rhythmic auditory stimulation has yielded improvements in vocal control, jitter, and shimmer in PD patients [52]. These interventions enhance engagement and satisfaction, suggesting potential applications beyond motor function. Haptic biofeedback systems have also shown promise in addressing dysphagia, offering non-invasive, real-time tactile stimuli that improve swallowing frequency and quality of life [51].

### 3.3. Effects of HRV Biofeedback on Autonomic Regulation and Emotional Function

Heart rate variability (HRV) biofeedback interventions target autonomic dysfunction and emotional dysregulation, which are common non-motor symptoms in PD. These systems typically use wearable sensors to deliver real-time feedback on heart rate and breathing patterns, enabling patients to enhance parasympathetic activity and autonomic balance. HRV biofeedback has demonstrated efficacy in reducing blood pressure, lowering stress markers, and improving mood and quality of life. Studies report reductions in depressive symptoms, enhanced patient-reported outcomes [40,49], and increased EEG complexity following motor imagery-based neurofeedback, albeit in elderly rather than PD populations [47]. Its feasibility for home-based training further supports its utility in long-term self-regulated rehabilitation.

### 3.4. Clinical Application Models for Biofeedback Integration in Parkinson’s Disease Rehabilitation

In light of these advancements, several clinical application models have been proposed to facilitate the effective integration of biofeedback and neurofeedback technologies into routine rehabilitation strategies for Parkinson’s disease. The translation of biofeedback and neurofeedback technologies into effective clinical practice models represents a crucial next step in Parkinson’s disease (PD) rehabilitation. Several frameworks can be envisioned to optimize their integration. Multidisciplinary rehabilitation protocols can combine physiotherapy, speech therapy, and occupational therapy with personalized biofeedback modules to simultaneously address motor and non-motor symptoms. For instance, wearable EMG biofeedback devices may be employed during gait and balance retraining, while EEG-based neurofeedback strategies can support cognitive rehabilitation efforts targeting executive function and attentional control [45]. Emerging multimodal frameworks, such as those combining EEG, HRV, and photoplethysmography (PPG), have shown potential in addressing both motor and non-motor domains, including depressive symptoms and gait stability [49]. Home-based biofeedback systems, which are increasingly becoming feasible through smartphone applications and wearable sensors, offer an opportunity to extend training beyond the clinic. Remote biofeedback programs can facilitate continuous monitoring, adaptive feedback delivery, and telerehabilitation supervision, promoting patient autonomy while reducing healthcare system burdens. In addition, hybrid neuromodulation strategies that combine biofeedback with non-invasive brain stimulation techniques, such as repetitive transcranial magnetic stimulation (rTMS) or transcranial direct current stimulation (tDCS), may yield synergistic effects [50]. In such models, initial neuromodulatory priming of cortical excitability may be followed by feedback-driven motor or cognitive training to reinforce adaptive neuroplasticity. Several clinical studies support these application models. For example, wearable EMG biofeedback has improved gait and balance in PD patients [45], while multimodal systems incorporating EEG, HRV, and PPG feedback have demonstrated combined benefits on mood and mobility [49]. Synergistic interventions pairing EEG neurofeedback with rTMS have shown significant improvements in both motor function and quality of life [50].

The personalization of biofeedback interventions through artificial intelligence and machine learning represents another promising avenue. By dynamically adapting training parameters to individual patient profiles and real-time performance data, AI-enhanced biofeedback systems can maximize the therapeutic efficacy and long-term retention of functional gains. Successful clinical translation will also require investment in interdisciplinary education and professional training. Standardized certification programs and clinical guidelines should be developed to ensure that rehabilitation specialists are proficient in implementing and monitoring biofeedback-based interventions safely and effectively.

### 3.5. Trends and Clinical Implications

The reviewed studies reveal modality-specific trends: EMG biofeedback predominantly improves motor control, HRV biofeedback addresses autonomic and emotional symptoms, and novel interfaces such as haptic and VR-integrated feedback show cross-domain potential [44,45,51,52]. Emerging multimodal approaches combining EEG, EMG, and HRV feedback may offer synergistic benefits across motor and non-motor domains [49]. Specifically, EEG neurofeedback has shown promising results in enhancing both motor symptoms and cognitive functioning, particularly when combined with neuromodulation techniques such as rTMS [50], and even in non-clinical populations through SMR protocols that improve attention and event-related potentials [43]. The clinical feasibility varies, with EMG and HRV systems being more accessible and suited for home-based use, while EEG biofeedback requires specialized infrastructure. These distinctions underscore the importance of personalized rehabilitation strategies tailored to specific functional targets in PD.

### 3.6. Methodological Constraints in Reviewed Studies

To provide a clearer overview of the research limitations identified in the reviewed studies, we have included a dedicated column (“Key Limitations”) in Table 1. This column highlights the principal methodological constraints encountered across the literature, which are crucial for interpreting outcomes and guiding future research. These include:

- Small sample sizes, often below the threshold for statistical power, which limit the generalizability of findings.
- Short intervention durations and lack of long-term follow-up, which restrict the ability to assess sustained effects of biofeedback interventions.
- Heterogeneity in populations, including studies with mixed cohorts (e.g., PD and stroke), or the use of healthy controls rather than PD patients.
- Limited use of control groups and randomization, reducing internal validity.
- Absence of blinding, particularly in pilot or feasibility studies, increasing risk of bias.
- Variability in outcome measures, which hinders cross-study comparisons.
- Focus on feasibility or acceptability over efficacy, especially in studies involving novel technologies (e.g., VR- or AI-driven biofeedback).

These issues highlight the need for standardized research protocols, larger randomized controlled trials, and more consistent outcome reporting. Such methodological improvements will be essential to strengthen the evidence base and support the clinical translation of biofeedback and neurofeedback interventions in Parkinson’s disease rehabilitation.

## 4. Discussion

The review reveals both opportunities and challenges in integrating biofeedback into PD care. While multiple modalities offer benefits, their translation into standard practice is contingent upon addressing key limitations related to standardization, accessibility, and personalization. This review underscores key opportunities and challenges in implementing biofeedback and neurofeedback in the clinical management of Parkinson’s disease (PD). Among the available modalities, EMG-based biofeedback stands out for its clinical feasibility. Due to its low cost, portability, and user-friendliness, EMG-based biofeedback is widely accessible in physiotherapy and neurorehabilitation settings and can be administered with minimal specialized training [53,55]. Similarly, HRV biofeedback systems, using simple wearable sensors (e.g., chest straps and finger pulse oximeters), are increasingly accessible for both clinic-based and home-based use, which can be facilitated by smartphone apps and remote monitoring capabilities [56].

In contrast, EEG-based neurofeedback, while promising in modulating cortical oscillations, presents considerable implementation challenges. It requires a controlled environment, real-time signal processing, and professional supervision, making it more suitable for research or specialized centers [36,57]. Although consumer-grade EEG devices are emerging, their clinical validity is still under scrutiny [38,58]. EEG-based neurofeedback interventions in PD primarily target pathological brain oscillations, such as excessive beta-band synchronization (linked to bradykinesia and rigidity) [59] and diminished frontal theta activity (associated with cognitive dysfunction) [60]. By training patients to down-regulate beta power or enhance frontal–midline theta rhythms, these interventions aim to restore functional neural dynamics. Several studies have reported that reductions in beta activity correlate with improved motor initiation and reduced rigidity, while increased theta power is associated with enhanced executive function and attentional control [61,62]. However, the consistency of these effects varies, with some trials demonstrating more robust gains than others, likely due to differences in protocol design, feedback thresholds, and patient engagement. Thus, while EEG neurofeedback shows substantial potential, further research is needed to standardize methods and confirm efficacy across diverse patient populations [39,58,63].

Thus, EMG and HRV biofeedback currently offer the most scalable options, while EEG requires further technical development and validation. A major theme emerging from the review is the heterogeneity of the included studies, spanning sample sizes, intervention durations, feedback modalities, and outcome domains. This variability limits the ability to draw broad generalizations. However, sub-group trends were identified: EEG-based interventions were more often associated with cognitive and attentional gains [37]; EMG biofeedback was linked to improvements in motor control and movement precision [64,65]; and HRV biofeedback impacted autonomic regulation and emotional well-being [33,35].

Despite these promising modality-specific outcomes, the evidence base remains limited by methodological inconsistencies, small sample sizes, and variability in outcome reporting [66]. Several clinical and methodological challenges must be addressed for broader implementation. A critical issue is the lack of standardized protocols—interventions vary in session length, reinforcement mechanisms, and targeted physiological signals. Patient-specific factors, such as attention deficits or executive dysfunction, may also affect the ability to engage with feedback and limit training efficacy. Identifying biomarkers predictive of responsiveness can enable tailored interventions and optimize outcomes [67].

Another barrier is the integration of biofeedback with conventional pharmacological and neuromodulatory treatments. Synergistic applications, such as combining biofeedback with dopaminergic therapy, deep brain stimulation (DBS), or non-invasive brain stimulation, are theoretically compelling but empirically underexplored. A notable exception includes studies combining EEG neurofeedback with rTMS, showing synergistic improvements in motor and cognitive outcomes in PD [34,50].

Looking ahead, future directions include the development of AI-enhanced feedback systems that dynamically adapt to patient performance, improving engagement and therapeutic impact [36,67,68]. For example, commercial platforms such as NeuroCoach^®^ and Myndlift^®^ provide EEG-based neurofeedback training with machine learning algorithms that personalize the intervention process in real time, adjusting thresholds and feedback modalities based on user-specific progress. Research prototypes like Adaptive NeuroTrainer involve exploring similar adaptive mechanisms for optimizing neuroplasticity through feedback modulation. The growth of wearable and mobile platforms may allow for home-based, real-time monitored biofeedback, improving accessibility and long-term adherence. Devices such as the Muse™ EEG headband and Emotiv Insight™ offer users the opportunity to engage in structured brain training sessions from home, with cloud-based monitoring options that can support remote clinical supervision [38,58]. Similarly, HRV biofeedback systems like the HeartMath^®^ Inner Balance and Elite HRV platforms are already being used for autonomic regulation and stress management in both clinical and consumer settings. EMG wearables such as Myo^®^ and NeuroNode™ also provide emerging avenues for home-based motor training [65,69].

Finally, multimodal biofeedback frameworks, integrating EEG, EMG, and HRV, may yield synergistic effects across motor, autonomic, and cognitive domains, enabling a more holistic approach to PD management [70].

## 5. Study Limitations and Future Directions

This review is subject to several limitations that must be acknowledged. First, the number of studies included (*n* = 15) remains relatively small, and although stringent eligibility criteria were applied, the sample may not fully represent the breadth of biofeedback research in Parkinson’s disease (PD). Second, the included studies displayed substantial heterogeneity in terms of biofeedback modality, intervention duration, target outcomes (motor, autonomic, and cognitive), and methodological rigor. This variability limits the abilities to synthesize results quantitatively and perform meta-analytic comparisons. Third, the absence of a formal quality appraisal tool (e.g., GRADE and PEDro scale) prevents detailed evaluation of the internal validity of the included trials.

Some studies relied on pilot data and lacked long-term follow-up, restricting conclusions about the durability of treatment effects. Several interventions were also tested in highly specific contexts (e.g., swallowing and vocal training), which may limit generalizability. Finally, due to the narrative nature of this review, there may be unintentional selection bias, even though a systematic search strategy was implemented.

These limitations point to the need for future reviews to include broader literature databases, formal risk-of-bias assessments, and meta-analytical techniques when feasible. Future studies should prioritize standardized outcome measures, larger sample sizes, and longitudinal follow-up to clarify the efficacy and sustainability of biofeedback interventions in PD. Cross-comparisons between modalities (e.g., EEG vs. EMG) and patient-centered analyses (e.g., predictors of responsiveness) will be critical to refining personalized therapeutic models.

## 6. Conclusions

Biofeedback is emerging as a promising non-pharmacological strategy in the rehabilitation of Parkinson’s disease. By facilitating the self-regulation of physiological functions through real-time feedback, biofeedback interventions align closely with the principles of personalized medicine and patient-centered care. Among the modalities reviewed, EMG biofeedback shows particular promise in improving muscle activation, reducing rigidity, and enhancing motor coordination. HRV biofeedback offers benefits in managing autonomic dysfunctions, stress, and mood disturbances, which are key non-motor symptoms. EEG neurofeedback, although more complex to deploy, has demonstrated potential in improving executive function and cognitive flexibility.

Despite encouraging results, large-scale clinical adoption faces several barriers: heterogeneity in protocols, limited access to advanced biofeedback technologies, and variability in patient responsiveness. Addressing these issues will require standardization of intervention parameters, greater integration with other therapeutic modalities, and sustained research investment.

With advancements in artificial intelligence, wearable biofeedback devices, and telerehabilitation platforms, biofeedback can become a core component of comprehensive PD rehabilitation. Future models may integrate multimodal feedback, VR environments, or brain–computer interfaces, maximizing therapeutic potential through technologically augmented, neurophysiologically informed care.

This comprehensive review included a total of 15 peer-reviewed studies investigating various biofeedback and neurofeedback interventions for Parkinson’s disease. The selected literature provides emerging evidence for their effectiveness in improving both motor and non-motor symptoms.

## Figures and Tables

**Table 1:** Summary of biofeedback and neurofeedback interventions for motor and non-motor symptoms in Parkinson’s disease.

Authors (Year)		Sample	Type of Biofeedback	Intervention Duration	Main Outcomes	Key Limitations	Study Design
1	Mirelman et al. (2011) [40]	PD patients	Audio-based	6 weeks	↑ Posture, balance	Small sample; limited to short-term follow-up	Controlled trial
2	Nanhoe-Mahabier et al. (2012) [41]	20 PD patients	Vibrotactile	Single session	↓ Trunk sway; ↑ Balance	Single session; no control group	Pre–post experimental
3	Caudron et al. (2014) [42]	17 PD patients	Visual	Single session	↓ Postural bias; ↑ Orientation	Single session; lacks generalizability	Pre–post experimental
4	Kober et al. (2014) [43]	20 (10 exp/10 ctrl)	EEG (SMR)	10 sessions	↑ Attention, memory, ERP	Small sample size; healthy subjects only	RCT
5	Byl et al. (2015) [44]	20 PD + stroke patients	Visual (Gait)	8 weeks	↑ Gait parameters, motor control	Mixed population (PD and stroke); no long-term follow-up	Interventional study
6	Carpinella et al. (2017) [45]	42 PD patients	Sensor-based (Wearable)	20 sessions	↑ Balance, gait	Pilot RCT; short duration	Pilot RCT
7	Roskopf et al. (2019) [46]	PD patients	Vibrotactile	4 weeks	↓ Postural sway; ↑ Balance	Small sample; lack of blinded assessment	Controlled trial
8	Arone et al. (2021) [32]	6 PD patients	EMG (Swallowing)	18 sessions	↑ Swallowing retention	Very small sample; no randomization	Pilot study
9	Bowman et al. (2021) [26]	PD patients	Visual + Auditory	6 weeks	↑ Gait speed, step length	No blinding; limited sample	Randomized controlled trial
10	Marcos-Martínez et al. (2021) [47]	11 elderly subjects	Motor Imagery EEG	5 sessions	↑ EEG complexity, cognition	Elderly subjects only; no PD patients	Pilot study
11	McMaster et al. (2022) [48]	PD patients	Visual (Trunk lean)	1 week + follow-up	↓ Trunk lean; ↑ Gait	Short follow-up; no control group	Pilot study
12	Shi et al. (2023) [49]	21 PD patients	Multimodal (EEG, HRV, PPG)	5 sessions	↓ Depression; ↑ Balance, gait	Small sample; limited intervention sessions	Pilot study
13	Romero et al. (2024) [50]	40 PD patients	EEG + rTMS	8 sessions	↑ Motor symptoms, QoL	Limited follow-up; moderate sample size	Randomized controlled trial
14	Xu et al. (2024) [51]	20 PD patients	AI-driven Haptic	Pilot phase	↑ Swallowing freq; high acceptance	Pilot design; acceptability prioritized over efficacy	Pilot study
15	da Cruz et al. (2025) [52]	30 PD patients	VR + EMG + RAS	2 sessions (7 days apart)	↓ Vocal jitter/shimmer; ↑ Engagement	Short duration; limited to voice parameters	Controlled trial

Note. Arrows indicate the direction of change observed following the biofeedback intervention: ↑ = Improvement or increase in the respective outcome (e.g., ↑ balance, ↑ gait speed); ↓ = Reduction or decrease in the respective outcome (e.g., ↓ depression, ↓ postural sway).

## Data Availability

No new data were created or analyzed in this study. Data sharing is not applicable to this article.
